# Effects of cholesterol depletion on compartmentalized cAMP responses in adult cardiac myocytes

**DOI:** 10.1016/j.yjmcc.2010.11.015

**Published:** 2011-03

**Authors:** Shailesh R. Agarwal, David A. MacDougall, Richard Tyser, Sara D. Pugh, Sarah C. Calaghan, Robert D. Harvey

**Affiliations:** a Department of Pharmacology—MS318, University of Nevada School of Medicine, Reno, NV 89557, USA; b Institute of Membrane and Systems Biology, University of Leeds, Leeds LS2 9JT, UK

**Keywords:** AKAP, A kinase anchoring protein, βAR, beta adrenergic receptor, cAMP, cyclic adenosine monophosphate, Cav-3, caveolin-3, PKA, protein kinase A, CGP, CGP20712A, Epac2, exchange protein activated by cAMP, EPR, E-type prostaglandin receptor, FRET, fluorescence resonance energy transfer, IBMX, 3-isobutyl-1- methylxanthine, *I*_Ca-L_, L-type Ca^2+^ current, ICI, ICI118,551, Iso, isoproterenol bitartrate, MβCD, methyl-β-cyclodextrin, PKA, protein kinase A, PGE1, prostaglandin E1, cAMP compartmentation, Lipid rafts, Adult ventricular myocytes

## Abstract

β_1_-Adrenergic receptors (β_1_ARs) and E-type prostaglandin receptors (EPRs) both produce compartmentalized cAMP responses in cardiac myocytes. The role of cholesterol-dependent lipid rafts in producing these compartmentalized responses was investigated in adult rat ventricular myocytes. β_1_ARs were found in lipid raft and non-lipid raft containing membrane fractions, while EPRs were only found in non-lipid raft fractions. Furthermore, β_1_AR activation enhanced the L-type Ca^2+^ current, intracellular Ca^2+^ transient, and myocyte shortening, while EPR activation had no effect, consistent with the idea that these functional responses are regulated by cAMP produced by receptors found in lipid raft domains. Using methyl-β-cyclodextrin to disrupt lipid rafts by depleting membrane cholesterol did not eliminate compartmentalized behavior, but it did selectively alter specific receptor-mediated responses. Cholesterol depletion enhanced the sensitivity of functional responses produced by β_1_ARs without having any effect on EPR activation. Changes in cAMP activity were also measured in intact cells using two different FRET-based biosensors: a type II PKA-based probe to monitor cAMP in subcellular compartments that include microdomains associated with caveolar lipid rafts and a freely diffusible Epac2-based probe to monitor total cytosolic cAMP. β_1_AR and EPR activation elicited responses detected by both FRET probes. However, cholesterol depletion only affected β_1_AR responses detected by the PKA probe. These results indicate that lipid rafts alone are not sufficient to explain the difference between β_1_AR and EPR responses. They also suggest that β_1_AR regulation of myocyte contraction involves the local production of cAMP by a subpopulation of receptors associated with caveolar lipid rafts.

## Research Highlights

► Not all cAMP coupled receptors produce functional responses in adult cardiac myocytes. ► Functional responses correlate with receptors found in lipid rafts domains. ► Lipid raft receptors stimulate local cAMP production detected in PKA signaling domains. ► Non-lipid raft receptors produced cAMP detected outside of PKA signaling domains. ► Cholesterol depletion alters lipid raft dependent responses but not compartmentation.

## Introduction

The diffusible second messenger cAMP plays a central role in regulating many different aspects of cardiac function. This includes the changes in electrical and mechanical properties of the heart produced by β_1_-adrenergic receptor (β_1_AR) activation in response to sympathetic stimulation [1,2]. However, a number of different G-protein-coupled receptors are linked to the production of cAMP in cardiac myocytes, yet they do not all produce the same functional responses. The E-type prostaglandin receptors (EPRs) are a classic example. These receptors stimulate cAMP production in ventricular myocytes, but they do not elicit changes in electrical or mechanical activity [3–6]. This can be explained by the idea that activation of any given type of receptor does not necessarily produce a uniform increase in cAMP throughout the entire cell and that cAMP signaling can be compartmentalized [7].

Despite unequivocal evidence that compartmentation of cAMP signaling occurs, what is responsible for this behavior is not well understood. Differences in phosphodiesterase activity have been shown to be essential for explaining the disparity in cAMP levels that can exist between different subcellular compartments within the cell [8,9]. However, phosphodiesterase activity alone cannot explain why different membrane receptors produce different patterns of cAMP production. Perhaps the simplest explanation for this observation assumes that different cytoplasmic compartments are associated with distinct plasma membrane domains containing unique receptor populations.

The formation of cholesterol-dependent lipid rafts is hypothesized to be an important means of organizing the plasma membrane into discrete signaling domains [10,11]. This includes caveolae, which represent a subset of lipid rafts that are defined by the presence of the protein caveolin [12]. It is believed that some proteins can be concentrated in these cholesterol-rich regions of the plasma membrane through lipid–protein or protein–protein interactions [13]. This includes certain types of G-protein-coupled receptors [14]. Consistent with this hypothesis, disrupting lipid rafts and caveolae by depleting the membrane of cholesterol has been shown to alter signaling associated specifically with receptors believed to exist in those domains [15–18].

In adult cardiac myocytes, β_1_ARs are found in caveolar and non-caveolar fractions of the plasma membrane [19]. On the contrary, studies using other cell types suggest that at least some EPR subtypes are excluded from caveolar membrane fractions [20,21]. Furthermore, we previously demonstrated that β_1_ARs stimulate cAMP production in a localized subcellular compartment that includes caveolae, as well as a bulk cytoplasmic compartment that is believed to exclude caveolae [22]. Yet, cAMP produced by EPRs could only be detected in the bulk cytoplasmic compartment [4]. These observations suggest that inclusion or exclusion of receptors from caveolar lipid raft domains may be important for determining the subcellular compartment in which they stimulate cAMP production. In the present study, we tested this hypothesis by examining the effect that cholesterol depletion has on cAMP-dependent responses as well as cAMP production associated with β_1_AR and EPR stimulation in adult ventricular myocytes.

## Materials and methods

### Cell isolation and culture

Ventricular myocytes were isolated from the hearts of male Wistar rats (250–300 g) using a modification of the procedure previously described [23]. The protocol used was in accordance with the *Guide for the Care and Use of Laboratory Animals* as adopted by National Institutes of Health and approved by the Institutional Animal Care and Use Committee at the University of Nevada, Reno. Myocytes used for fluorescence resonance energy transfer (FRET) imaging experiments were resuspended and plated in minimum essential medium (MEM) containing insulin–transferrin–selenium (1×), bovine serum albumin (1 mg/ml), 2,3-butanedione monoxime (10 mM), and penicillin–streptomycin. After incubation for 2 h, the cells were transduced with adenovirus constructs containing protein kinase A (PKA)- or Epac2-based cAMP biosensors, as described previously [4,22,24]. Imaging experiments were conducted 48–72 h after infection. Except where noted, all other experiments were carried out using myocytes resuspended in extracellular solution containing (in mM) NaCl 137, KCl 5.4, MgCl_2_ 0.5, CaCl_2_ 1.0, NaH_2_PO_4_ 0.33, HEPES 5, glucose 5.5, pH 7.4, and used on the day of isolation. All experiments were conducted at room temperature.

### Electrophysiology

Electrophysiology experiments were carried out using K^+^-free extracellular solution in which KCl was replaced with CsCl. Whole-cell currents were recorded using a Multiclamp 700B amplifier, Digidata 1440A digitizer, and pClamp software (Axon Instruments). Microelectrode resistances were between 1 and 2 MΩ when filled with intracellular solution containing (in mM) CsCl 130, TEA-Cl 20, EGTA 5, MgATP 5, TrisGTP 0.06, and HEPES 5 (pH 7.2). The voltage-clamp protocol employed a holding potential of −80 mV. A 50 ms pre-pulse to −40 mV to inactivate Na^+^ channels was followed by 100 ms test pulse to 0 mV to activate the L-type Ca^2+^ current (*I*_Ca-L_). Changes in *I*_Ca-L_ magnitude were monitored by repeating this protocol once every 5 s.

### FRET imaging

Experiments were carried out using intact myocytes expressing PKA- or Epac2-based biosensors, as described previously [4,22,24]. Changes in cAMP activity were defined as relative changes in the ratio of the background and bleed-through corrected CFP and YFP (FRET) fluorescence intensity measured over the area of the entire cell.

### Measurement of cell length and [Ca^2+^]

Myocyte shortening and [Ca^2+^]_i_ transients were measured simultaneously in myocytes loaded with fura-2AM, as described previously [25]. Cells were field stimulated at 0.5 Hz.

### Methyl-β-cyclodextrin (MβCD) treatment

Membrane cholesterol was depleted, as previously described [23], by suspending cells in extracellular solution containing 1 mM MβCD for 1 h at 37 °C. For FRET imaging experiments, transduced cells were incubated in the MEM-based culture medium containing 1 mM MβCD for 1 h at 37 °C.

Filipin, a polyene antibiotic that forms complexes with cholesterol that can be visualized by fluorescence microscopy, was used to verify that MβCD treatment effectively depleted membrane cholesterol [26]. Control or MβCD-treated cells were fixed in 3.7% formaldehyde in phosphate-buffered saline (PBS) for 10–15 min. Fixed cells were spun onto microscope slides using a Shandon Cytospin. After rinsing, cells were stained with PBS containing 0.05 mg/ml filipin (Sigma-Aldrich) and 10% fetal bovine serum (Hyclone). Fluorescence images were obtained using a D350/50x excitation filter, E420LP dichroic mirror, and a 400 DCLP emission filter.

### Membrane fractionation experiments

Membranes prepared from isolated myocytes were subjected to ultracentrifugation using a discontinuous sucrose density gradient [27]. Western blotting was then used to identify β_1_AR, as well as EP_2_, and EP_4_ receptor proteins separated by SDS-PAGE. Caveolar and extra-caveolar membrane fractions were identified by blotting for caveolin 3 (Cav-3) the muscle specific caveolin, and β-adaptin, respectively.

### Materials

Prostaglandin E1 (PGE1, Cayman Chemicals) and ICI 118,551(Tocris Bioscience) were prepared as stock solutions in DMSO. CGP 20712A (Tocris Bioscience) and isoproterenol bitartrate (Sigma-Aldrich) were prepared as stock solutions in water. Isoproterenol and 3-isobutyl-1-methylxanthine (Sigma-Aldrich) containing solutions were prepared fresh daily. Fura-2AM was purchased from Molecular Probes. β_1_AR, EP_2_R, and EP_4_R antibodies were obtained from Santa Cruz Biotechnology. β-Adaptin and Cav-3 antibodies were from BD Transduction Laboratories.

### Statistics

All results are expressed as the mean ± SEM of the results obtained from *n* number of cells. Statistical significance between two groups was defined by Student's *t-*test, *p* values of < 0.05. For comparison of more than 2 groups, 1-way ANOVA was used, with Holm-Sidak post hoc analysis.

## Results

**Fig. 1:**
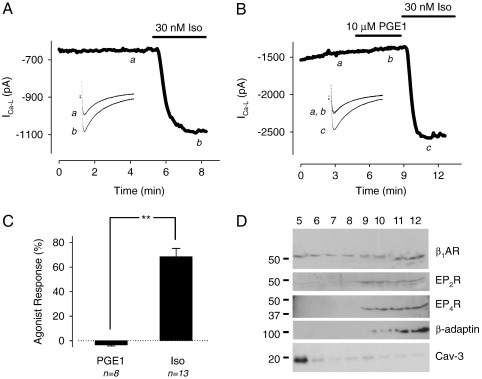
Effect of the β-adrenergic receptor (βAR) agonist isoproterenol (Iso) and the E-type prostaglandin receptor (EPR) agonist PGE1 on the L-type Ca^2+^ current (*I*_Ca-L_) in rat ventricular myocytes. A, Time course of changes in *I*_Ca-L_ amplitude and corresponding sample current traces (inset) under control conditions (a) and following exposure to 30 nM Iso (b). B, Time course of changes in *I*_Ca-L_ amplitude and corresponding sample current traces (inset) under control conditions (a), and following exposure to 10 μM PGE1 (b) and 30 nM Iso (c). C, Average increase in *I*_Ca-L_ amplitude recorded in the presence of 10 μM PGE1 or 30 nM Iso (**p < 0.05). D, Western blot of ventricular myocyte membrane fractions obtained by sucrose density centrifugation. Caveolin-3 (Cav-3) was used as a marker of caveolae-containing buoyant membranes (fraction 5); β-adaptin was used as a marker of heavy non-caveolar membranes (fractions 9–12).

It has previously been shown that the βAR agonist isoproterenol (Iso) and the EPR agonist prostaglandin E1 (PGE1) both stimulate cAMP production in adult cardiac myocytes, but only Iso enhances the L-type Ca^2+^ current (*I*_Ca-L_) [4,5]. The differences in the effects of these agonists on the *I*_Ca-L_ are illustrated in Fig. 1A and B. To determine whether or not this disparity in functional responses correlates with a difference in where the corresponding receptors are found in the plasma membrane, we conducted membrane fractionation experiments. Membranes prepared from isolated myocytes were subjected to ultracentrifugation using a discontinuous sucrose density gradient. Immunoblotting was then used to identify β_1_ARs, as well as EP_2_, and EP4 receptors, the EPR subtypes associated with cAMP production [28]. The results (Fig. 1D) indicate that the β_1_AR can be detected in all fractions, including the caveolar fraction (5), which is identified by its enrichment in the caveolar protein, caveolin-3. The results also demonstrate that EP_2_ and EP_4_ receptors are expressed in these cells. However, unlike the β_1_AR, the EP receptors are excluded from caveolar membrane fractions.

**Fig. 2:**
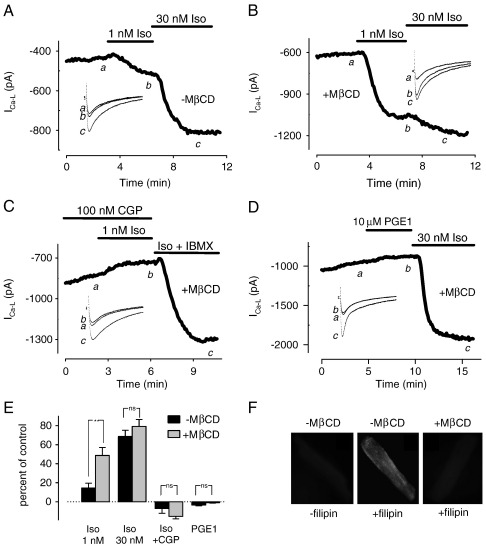
Effect of cholesterol depletion on L-type Ca^2+^ current (*I*_Ca-L_) responses to isoproterenol (Iso) and PGE1. Time courses of change in *I*_Ca-L_ amplitude and corresponding sample current traces (inset). A, Untreated cell under control conditions (a), and following exposure to 1 nM (b) and 30 nM (c) Iso. B, MβCD-treated cell under control conditions (a), and following exposure to 1 nM (b) and 30 nM (c) Iso. C, MβCD-treated cell in the presence of the selective β_1_-adrenergic receptor antagonist CGP20712A (CGP, 100 nM) (a), and following exposure to CGP plus 1 nM Iso (b) and 1 μM Iso plus the phosphodiesterase inhibitor 3-isobutyl-1-methylxanthine (IBMX, 100 μM) (c). D, MβCD-treated cell under control conditions (a), and following exposure to 10 μM PGE1 (b) and 30 nM Iso (c). E, Average change in *I*_Ca-L_ amplitude in untreated and MβCD-treated myocytes (**p < 0.05, ns = not significant, see text for *n* numbers). F, Effect of MβCD treatment on membrane cholesterol content detected by filipin staining.

In neonatal ventricular myocytes, cholesterol depletion has been shown to have no effect on the *I*_Ca-L_ response to maximal β_1_AR stimulation [29]. However, there are significant differences between neonatal and adult ventricular myocytes that can affect compartmentation of cAMP signaling [19,30]. Therefore, we examined the effect of cholesterol depletion on the *I*_Ca-L_ response to β_1_AR stimulation in adult myocytes. To do this, we measured the effects produced by Iso at sub-maximally (1 nM) and maximally (30 nM) stimulating concentrations. In control cells, 1 nM Iso increased the *I*_Ca-L_ by 15 ± 5.0% (*n* = 13) over baseline. Subsequent exposure to 30 nM Iso increased the current by 69 ± 6.6% (*n* = 13) (Fig. 2A and E).

This experiment was then repeated in cells pretreated with 1 mM methyl-β-cyclodextrin (MβCD) for 1 h at 37 °C. This approach has been used previously to deplete membrane cholesterol and disrupt lipid rafts and caveolae in cardiac myocytes [23,29,31]. Consistent with this, MβCD treatment significantly reduced the membrane cholesterol content as detected by staining with the fluorescent polyene antibiotic filipin (Fig. 2F). Furthermore, when MβCD-treated cells were exposed to 1 nM Iso, the *I*_Ca-L_ increased to 49 ± 8.3% (*n* = 5) over baseline. This represents a 3.2-fold increase in the magnitude of the response to this sub-maximally stimulating concentration of Iso compared with that seen in control cells. Exposure to 30 nM Iso increased the current by 79.2 ± 7.2% (*n* = 6) over baseline (Fig. 2B and E), which was not significantly different from the response observed in control cells.

Rat ventricular myocytes express both β_1_ and β_2_ARs, and both receptor subtypes have been reported to stimulate L-type Ca^2+^ channel activity in these cells [32]. To verify that the consequence of MβCD treatment was due to an effect on β_1_AR responses, we repeated the above experiments in the presence of 100 nM CGP20712A (CGP), a selective β_1_AR antagonist [33]. In cells pretreated with 100 nM CGP, subsequent exposure to 1 nM Iso produced no response (*n* = 4) (Fig. 2E). CGP also blocked the effect of 1 nM Iso in MβCD-treated cells (*n* = 5) (Fig. 2C and E). As a positive control, subsequent exposure to the phosphodiesterase inhibitor IBMX was used to elicit a maximal response. These results support the conclusion that cholesterol depletion enhances *I*_Ca-L_ sensitivity to β_1_AR stimulation.

**Fig. 3:**
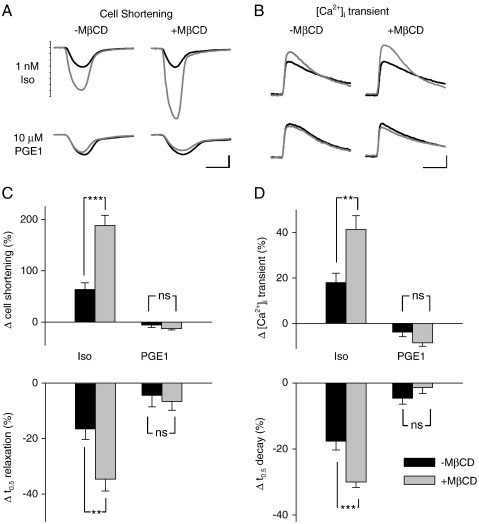
Effect of cholesterol depletion on intracellular Ca^2+^ ([Ca^2+^]_i_) transient and cell shortening responses to isoproterenol (Iso) and PGE1. A, Cell shortening elicited by electric field stimulation before (black traces) and after (gray traces) exposure to 1 nM Iso (upper panels) and 10 μM PGE1 (lower panels) in untreated cells (left hand panels) and MβCD-treated cells (right hand panels). Scale bars: 200 ms, 4% resting of cell length. B, [Ca^2+^]_i_ transients elicited by electric field stimulation before (black traces) and after (gray traces) exposure to 1 nM Iso (upper panels) and 10 μM PGE1 (lower panels) in untreated cells (left hand panels) and MβCD-treated cells (right hand panels). Scale bars: 200 ms, 0.4 relative units. C, Average cell shortening responses expressed as the change relative to resting cell length in untreated and MβCD-treated cells (upper panel); average change in time to half (*t*_0.5_) relaxation of cell shortening in control and MβCD-treated cells (lower panel). D, Average [Ca^2+^]_i_ transient responses expressed as the change in fura-2 fluorescence ratio relative to baseline in untreated and MβCD-treated cells (upper panel); average change in *t*_0.5_ decay of the [Ca^2+^]_i_ transient in control and MβCD-treated cells (lower panel). Responses to 1 nM Iso were recorded in the presence of 100 nM ICI 118,551, a selective β_2_ receptor antagonist (**p < 0.01, ***p < 0.001, ns = not significant; see text for *n* numbers).

It has been reported that L-type Ca^2+^ channels are found in the caveolar membrane fraction of cardiac myocytes [29,34]. To determine whether or not the effect of cholesterol depletion was specific for β_1_AR regulation of the *I*_Ca-L_, we also examined the effect of MβCD treatment on the intracellular Ca^2+^ ([Ca^2+^]_i_) transient and myocyte contraction (Fig. 3). In these experiments, myocytes were exposed to 1 nM Iso in the presence of 100 nM ICI 118,551 (ICI), a β_2_AR antagonist, to selectively activate β_1_ARs [33]. As expected, this protocol resulted in a significant increase in the magnitude of the [Ca^2+^]_i_ transient and myocyte shortening in untreated cells. Following exposure to Iso, the relative degree of cell shortening increased by 63 ± 13% and the magnitude of the [Ca^2+^]_i_ transient increased by 18 ± 4.1% (*n* = 17). These effects were also accompanied by a significant increase in the rate with which both parameters returned to baseline.

The half-time (*t*_0.5_) for relaxation of cell shortening decreased by 17 ± 3.8% and the *t*_0.5_ for decay of the [Ca^2+^]_i_ transient decreased by 18 ± 2.6%. When these experiments were repeated in MβCD-treated myocytes, the response to 1 nM Iso was significantly enhanced. The relative degree of cell shortening increased by 188 ± 19.5% and the magnitude of the [Ca^2+^]_i_ transient increased by 35 ± 4.3% (*n* = 22). There were also significant changes in the rate that both parameters returned to baseline. The *t*_0.5_ for relaxation of cell shortening decreased by 35 ± 4.3% and the *t*_0.5_ for decay of the [Ca^2+^]_i_ transient decreased by 30 ± 1.7%.

Next we examined the effect of MβCD treatment on responses to PGE1. If the effect of cholesterol depletion is a non-specific effect altering all receptor-mediated cAMP-dependent responses, then it is conceivable that this treatment might also alter the response to PGE1, revealing functional effects not observed in control cells. Such an effect might be expected if compartmentation of PGE1 responses is due to exclusion of EP receptors from lipid raft domains, given that membrane fractionation studies suggest that cholesterol depletion causes a redistribution of membrane proteins involved in cAMP signaling [18,35,36]. However, neither of these possibilities appeared to be true. Exposure to PGE1 did not alter the *I*_Ca-L_ in either untreated (see Fig. 1) or MβCD-treated cells (see Fig. 2D and E). Consistent with this, PGE1 did not significantly affect the [Ca^2+^]_i_ transient or myocyte shortening in either untreated or MβCD-treated cells (see Fig. 3). These results suggest that the effect of MβCD treatment and cholesterol depletion was not a non-specific effect, since it selectively altered responses produced by β_1_AR stimulation.

Evidence presented here and elsewhere indicates that in cardiac myocytes, β_1_ARs can be found in all membrane fractions, not just those associated with caveolin [19,29,35]. This raises the question of whether or not the change in sensitivity to β_1_AR signaling caused by cholesterol depletion is due to an effect on those receptors specifically associated with caveolar lipid raft domains. To address this question, we examined the effect of cholesterol depletion on β-adrenergic stimulation of cAMP production in intact myocytes using different FRET-based biosensors.

**Fig. 4:**
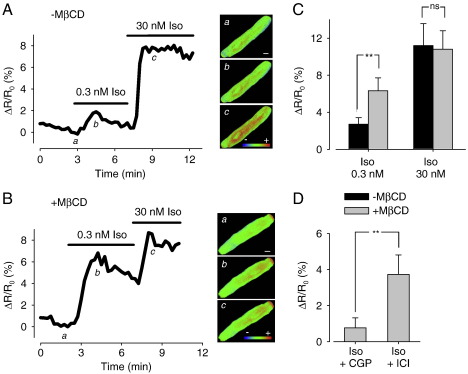
Effect of cholesterol depletion on intracellular cAMP response to β-adrenergic receptor stimulation detected by the type II PKA biosensor. Time course of changes in FRET response (Δ*R*/*R*_0_) and corresponding pseudocolor images recorded under control conditions (a), and following exposure to 0.3 nM (b) and 30 nM Iso (c) in an untreated cell (A) and a MβCD-treated cell (B). Scale bar, 10 μm. C, Average changes in FRET responses in untreated and MβCD-treated cells. D, Average changes in FRET response to 0.3 nM Iso in MβCD-treated cells recorded in the presence of the β_1_ receptor antagonist CGP20712A (CGP, 100 nM) or the β_2_ receptor antagonist ICI 118,551 (ICI, 100 nM) (**p < 0.05, ns = not significant).

In the first set of experiments, we used a PKA-based biosensor (Fig. 4) [4,24]. This particular probe contains the type II regulatory (RII) subunit of PKA [37]. Because of this, it is targeted to specific subcellular locations through its interactions with A kinase anchoring proteins (AKAPs) [4,37]. This includes caveolar fractions of the plasma membrane [29,34,35]. Therefore, it is expected to respond to changes in cAMP that occur in microdomains that include caveolar lipid raft domains [38]. Consistent with this idea, we have previously demonstrated that it detects cAMP responses that are distinctly different from those occurring in the total cytosolic cAMP compartment [4,22].

In control myocytes expressing the PKA-based biosensor, exposure to 0.3 nM Iso produced a 2.7 ± 0.7% change in the FRET response (*n* = 7). Subsequent exposure to 30 nM Iso increased the FRET response to 11.2 ± 2.4% of control (*n* = 7) (Fig. 4A and C). When these experiments were repeated in MβCD-treated myocytes, 0.3 nM Iso produced a 6.3 ± 1.4% change in the FRET response (*n* = 6), which increased to 10.8 ± 2.0% (*n* = 6) following subsequent exposure to 30 nM Iso (Fig. 4B and C). These results indicate that cholesterol depletion caused a significant increase in the sensitivity of the response to sub-maximally, but not maximally stimulating concentrations of Iso. Furthermore, these effects were blocked in the presence of CGP, but not ICI (Fig. 4D), confirming that the increase in sensitivity of the PKA-based biosensor's response to Iso caused by cholesterol depletion was due to an effect on β_1_AR signaling.

It is unlikely that the effects of cholesterol depletion are due to disruption of AKAP binding causing the redistribution of type II PKA and the PKA-based biosensor. This is because disrupting AKAP targeting of type II PKA has been shown to diminish β-adrenergic regulation of L-type Ca^2+^ channels [39], which is opposite to the enhanced response we observed following cholesterol depletion. Furthermore, the PKA-based biosensor is expressed in a striated pattern along T tubules in adult ventricular myocytes [24], and we previously demonstrated that blocking PKA anchoring to AKAPs disrupts this pattern [4]. However, when we examined the expression pattern in a similar manner following MβCD treatment, we found no change (see Supplementary Fig. S1).

Because these experiments involved the use of cells maintained in culture for up to 72 h, we also examined the effect that this had on their cholesterol content and found that there was a time dependent decrease. After 72 h, the cholesterol content, as determined by filipin staining, was reduced by > 50% (see Supplementary Fig. S2). Nevertheless, the response observed in MβCD-treated cells was consistent with an effect due to depletion of the remaining cholesterol. This is supported by the fact that treatment with α-cyclodextrin, an analog of MβCD that does not bind cholesterol [40], had no effect on PKA probe response to β_1_AR stimulation (see Supplementary Fig. S3).

**Fig. 5:**
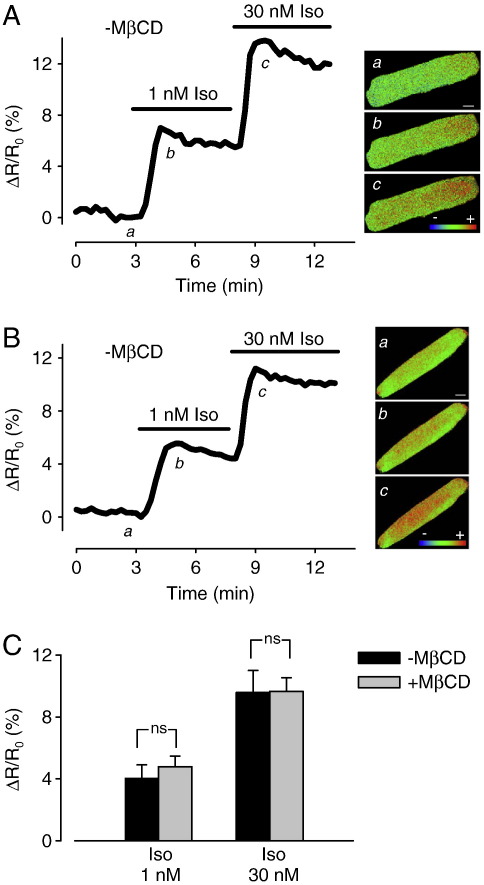
Effect of cholesterol depletion on intracellular cAMP response to β-adrenergic receptor stimulation detected by the Epac2 biosensor. Time course of changes in FRET response (Δ*R*/*R*_0_) and corresponding pseudocolor images under control conditions (a), and following exposure to 1 nM (b) and 30 nM Iso (c) in an untreated cell (A) and a MβCD-treated cell (B). Scale bar, 10 μm. C, Average change in FRET responses in untreated cells and MβCD-treated cells (ns = not significant).

In the next set of experiments, we examined the effect of cholesterol depletion on cAMP responses detected using Epac2-camps (Fig. 5), a biosensor created using one of the cAMP binding domains from the type 2 exchange protein activated by cAMP [41]. Because it lacks any of the anchoring sequences found in the full-length protein, it is expressed uniformly throughout the cytoplasm of the cell. As a result, it is able to respond to changes in total cellular cAMP [22]. We have previously demonstrated that the responses detected by this Epac2-based biosensor are distinctly different from those detected by the PKA-based biosensor [4]. In control myocytes expressing the Epac2 probe, exposure to 1 nM Iso produced a 4.1 ± 0.8% change in the FRET response. This increased to 10.2 ± 1.4 % upon exposure to 30 nM Iso (*n* = 6). When this experiment was repeated in MβCD-treated cells, there was no significant change in the response to either concentration of Iso. The FRET responses to 1 nM and 30 nM Iso were 4.8 ± 0.7% and 9.7 ± 0.9%, respectively (*n* = 5). These results suggest that the Epac2 probe is more sensitive to cAMP produced by β_1_ARs found outside of lipid raft domains.

**Fig. 6:**
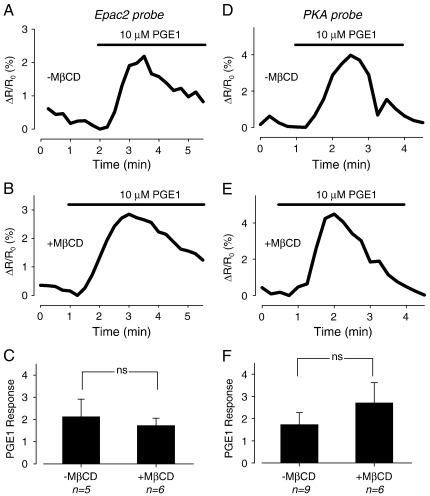
Effect of cholesterol depletion on intracellular cAMP response to PGE1. Time course of FRET response detected by Epac2-based biosensor (A, B) or type II PKA-based biosensor (D, E) following exposure to 10 μM PGE1 in untreated cells (A, D) and MβCD-treated cells (B, E). Average changes in FRET response to 10 μM PGE1 detected by Epac2-based biosensor (C) and PKA-based biosensor (F) in untreated and MβCD-treated cells (ns = not significant).

In a final set of experiments, we examined the effect of PGE1 on cAMP responses detected by both FRET-based biosensors. We have previously demonstrated that PGE1 is able to elicit a transient increase in cAMP that is detected by the Epac2 probe, but not the PKA probe in adult guinea pig ventricular myocytes [4]. Consistent with this, we found that exposure to 10 μM PGE1 produced a similar transient increase in cAMP that was detected by the Epac2 probe in adult rat ventricular myocytes (Fig. 6A). This confirms that PGE1 is indeed capable of stimulating cAMP production in these cells. Exposure to PGE1 also produced a transient response that was detected by the Epac2 probe in MβCD-treated cells (Fig. 6B). However, the magnitude of the response was not significantly different from what was observed in control cells (Fig. 6C). Surprisingly, when these experiments were repeated in myocytes expressing the PKA probe, PGE1 treatment still elicited a transient change in FRET response (Fig. 6D). However, the magnitude of the transient response was not significantly different between control and MβCD-treated myocytes (Fig. 6E and F).

The ability of PGE1 to stimulate cAMP production, even though it had no effect on functional responses, supports the idea that the cAMP produced by PGE1 is compartmentalized. However, because the cAMP responses were measured in cells maintained in culture, it is possible that changes in their composition or structure could have created this apparent discrepancy. To address this possibility, we looked at the effect of 10 μM PGE1 on the *I*_Ca-L_ current in cells kept in primary culture for 72 h. Just as in freshly isolated cells, PGE1 had no effect on the *I*_Ca-L_, despite the fact that subsequent exposure to 30 nM Iso produced a normal response (see Supplementary Fig. S4). These results suggest that compartmentation of cAMP signaling is not altered by any changes that may have occurred with time in culture.

## Discussion

The observation that two agonists can differ in their capacity to elicit PKA-dependent responses, despite the fact that they both stimulate cAMP production, cannot be easily reconciled if it is assumed that the receptors involved are distributed uniformly throughout the plasma membrane. In the present study, we examined the role that differences in the distribution of β_1_ARs and EPRs in cholesterol-dependent lipid rafts may play in contributing to the disparities in functional responses they produce. Previous studies have used biochemical methods to demonstrate that caveolar lipid rafts are associated with the segregation of various G-protein-coupled receptors [14], but most studies involving cardiac myocytes have relied on the use of neonatal cells [21,29,35,42], and none have looked directly at the role that lipid rafts play in explaining differences in functional responses in adult myocytes. Because neonatal myocytes exhibit significant differences in structure and cell signaling responses [43,44], we chose to conduct our studies using adult ventricular myocytes. To gain further insight into the role of lipid rafts in generating compartmentalized responses by both β_1_ARs and EPRs, we also used live cell imaging of FRET-based biosensors to measure cAMP responses in different microdomains of intact myocytes.

The results of the present work indicate that even though both β_1_ARs and EPRs were able to stimulate cAMP production, as detected by FRET-based biosensors, only β_1_ARs were able to enhance the *I*_Ca-L_, [Ca^2+^]_i_ transient and myocyte shortening. Furthermore, we observed that β_1_ARs are found in caveolar as well as extra-caveolar fractions of the plasma membrane, while EPRs were only found in extra-caveolar fractions. When myocytes were then treated with MβCD to deplete cholesterol and disrupt lipid rafts, β_1_AR sensitivity of functional responses was enhanced. Consistent with this observation, MβCD treatment also enhanced β_1_AR sensitivity of the PKA probe response. However, MβCD treatment had no effect on the Epac2 probe response to β_1_AR stimulation, and it had no effect on any response to PGE1. These results support the idea that segregation of specific G-protein-coupled receptors within the membrane is an underlying mechanism of separation of function associated with different cAMP signaling pools. They also suggest that β_1_-adrenergic regulation of the functional responses described in this study is specific to receptors found in caveolar membrane domains.

The fact that MβCD treatment did not alter the PKA probe response to PGE1 suggests that the effect of cholesterol depletion was not due to a direct effect on endogenous PKA or the PKA probe. The more likely explanation for our results is that cholesterol depletion increased myocyte sensitivity to β_1_AR stimulation of cAMP production. This conclusion is consistent with biochemical studies reporting that cholesterol depletion increases βAR stimulation of cAMP production in various cell types [15,35,36]. Rybin et al. [35] suggested that this can be explained by a direct interaction between adenylyl cyclase and the inhibitory scaffolding domain of caveolin-3. They concluded that cholesterol depletion disrupts this interaction, relieving its inhibitory effect on cAMP production. Removal of such an inhibitory effect could then explain the shift in sensitivity to β_1_AR stimulation that we observed.

In another study using neonatal rat ventricular myocytes, Ostrom et al. [18] reported that cholesterol depletion actually inhibits βAR stimulation of cAMP production. The reason for the apparent discrepancy is unclear. However, they also found that EPR stimulation of cAMP production was unaltered. Their conclusion was that cholesterol depletion only affects responses associated with receptors found in caveolar lipid rafts, which is consistent with our results in intact adult myocytes. More recently, Balijepalli et al. [29] reported that cholesterol depletion had no affect on β_1_AR regulation of the *I*_Ca-L_ in neonatal mouse ventricular myocytes. Yet, their study only compared responses to maximal receptor activation. Consistent with that, our present work found that cholesterol depletion did not alter responses to maximal β_1_AR stimulation, it only increased responses to sub-maximal β_1_AR stimulation, indicating that cholesterol depletion was shifting the sensitivity to receptor activation. Our previous work has shown evidence of a trend for cholesterol depletion to enhance β_1_AR responses produced by 5 nM Iso, although the effects were not statistically significant [23]. The greater effect observed in the present study may be explained by our use 1 nM Iso, a concentration much further from that which produces maximal responses.

Our finding that cholesterol depletion increased β_1_AR sensitivity of responses detected by the PKA-based biosensor, but not the Epac2-based biosensor, suggests that the regulation of electrical and mechanical activity involves the production of cAMP in a microdomain associated with type II PKA by receptors found in cholesterol-dependent lipid rafts. It also suggests that β_1_AR stimulation of cAMP throughout the rest of the cell may be mediated by receptors that are found primarily outside of lipid rafts. These results, together with the fact that PGE1 still had no effect on functional responses following cholesterol depletion, demonstrate that while receptors in lipid raft and non-lipid raft domains can produce compartmentalized responses, compartmentalized behavior still exists even after lipid rafts have been disrupted. This implies that cholesterol depletion may not be causing significant redistribution of these receptors and that something else must contribute to their association with different cAMP signaling domains. Although this appears to be different from the effect that cholesterol depletion has on β_2_ARs [17], it is consistent with reports that cholesterol depletion has no effect or actually decreases the mobility of many plasma membrane proteins, possibly due to the existence and/or enhancement of other barriers, such as actin cytoskeleton or solid phase phospholipids [45].

Another important observation made in the present study is that cholesterol depletion not only enhanced the magnitude of the [Ca^2+^]_i_ transient and myocyte shortening responses to β_1_AR stimulation, it also affected the rate of transient decay and the rate of relaxation. While an increase in the magnitude of the *I*_Ca-L_ could conceivably explain the change in the magnitude of those other responses, it is unlikely to explain the change in their kinetics. The faster decay of the [Ca^2+^]_i_ transient most likely reflects an increase in the rate of Ca^2+^ uptake by the sarcoplasmic reticulum Ca^2+^ ATPase due to PKA-dependent phosphorylation of phospholamban [2]. Such an effect is also likely to contribute to the faster relaxation of myocyte shortening, although PKA- dependent phosphorylation of troponin I could also play a role. These observations indicate that β_1_AR regulation of myocyte contraction correlates more closely with the local production of cAMP by a subpopulation of receptors associated with lipid rafts and that it is not just L-type Ca^2+^ channels that are under the local control of cAMP production by β_1_ARs.

The present study also demonstrates some interesting new aspects of the compartmentalized response associated with EP receptor activation. In the original study using isolated adult cardiac myocytes, Buxton and Brunton [3] employed a biochemical approach and found that PGE1 stimulated cAMP production in the soluble fraction, but not the particulate fraction, of homogenized myocytes. Following the development of biosensors capable of measuring cAMP activity in intact cells, Rochais et al. [5] used an exogenously expressed, cyclic nucleotide gated (CNG) ion channel, but found that it was unable to detect a cAMP response produced by PGE1 in adult rat ventricular myocytes. However, in our previous study using adult guinea pig ventricular myocytes, we found that PGE1 elicited a transient increase in cAMP detected by the Epac2-based probe, yet it had no effect on the cAMP response detected by the type II PKA-based probe anchored by AKAPs [4]. Similar results were reported in neonatal rat ventricular myocytes, where PGE1 stimulation was found to produce a more significant cAMP response outside of the type II PKA signaling domain [46]. This is in contrast to the present study in adult rat ventricular myocytes, where we found that PGE1 produced a transient cAMP response detected by both the Epac2- and type II PKA-based probes. This apparent discrepancy may reflect developmental and/or species-dependent differences in the distribution of type II PKA. Despite the fact that PGE1 produced a response detected by the PKA probe, it had no effect on any of the functional properties studied. This suggests that in rat ventricular myocytes, the type II PKA signaling domain includes one or more subcellular compartments distinctly separate from those involved in regulating electrical or mechanical activity. The observation that EP_2_ and EP_4_ receptors are found in extra-caveolar fractions of the plasma membrane suggests that PGE1 is producing cAMP in a subset of the PKA signaling domain not associated with caveolar lipid rafts. Consistent with this idea, evidence for type II PKA has been found in both caveolar and extra-caveolar membrane fractions of cardiac ventricular myocytes [29,35]. The question remains as to whether or not all or only some of the extra-caveolar PKA sees the cAMP produced by PGE1.

## Potential limitations

Experiments involving the use of FRET-based biosensors were carried out in myocytes kept in culture for 48–72 h, while most functional experiments involved the use of freshly isolated cells. It is possible that changes in the composition or structure of the cultured cells could have altered some of the cAMP responses we measured. For example, we observed that cholesterol content decreased with time. Still, this did not alter the inability of PGE1 to regulate Ca^2+^ channel function, which confirmed that compartmentation of cAMP signaling occurs in cultured cells. It has also been reported that T tubule density decreases with time in culture [47]. In adult ventricular myocytes, L-type Ca^2+^ channels involved in the regulation of excitation contraction coupling are located in T tubules, and a loss of T tubules has been shown to attenuate the *I*_Ca-L_ response to βAR regulation [48]. However, we found that β_1_AR of the *I*_Ca-L_ current was unaffected in cultured cells. The fact that cholesterol depletion altered the sensitivity of β_1_AR stimulation of cAMP detected by the PKA probe is also unlikely to have been due to the fact that those experiments were conducted in cultured cells since cholesterol depletion had a similar effect on cAMP-dependent functional responses in freshly isolated cells. Even so, we cannot rule out a potential contribution of changes occurring in culture to at least some of our observations.

## Conclusions

The present results support the idea that cAMP responses produced by receptors found in lipid rafts domains are sensitive to cholesterol depletion, while responses produced by receptors found outside of lipid raft domains are not. Based on this conclusion, the effects of cholesterol depletion suggest that β_1_ adrenergic regulation of electrical and mechanical responses in adult ventricular myocytes is mediated by those receptors associated specifically with lipid raft domains. Finally, even though cholesterol-dependent lipid rafts appear to be associated with compartmentalized cAMP responses, they alone are not sufficient to explain what is responsible for the difference between signaling domains associated with β_1_ adrenergic and EP receptors.
