# Supplementary material for: Effects of cholesterol depletion on compartmentalized cAMP responses in adult cardiac myocytes
Source: J Mol Cell Cardiol. 2011 Mar;50(3):500–9. doi: 10.1016/j.yjmcc.2010.11.015 (PMC3049871; doi:10.1016/j.yjmcc.2010.11.015)
Supplement: Supplementary file 1 — Supplementary material [file mmc1.pdf]

Supplementary Figures

Figure S1. Cholesterol depletion with methyl-β-cyclodextrin (MβCD) does not alter the expression pattern of the PKA-based biosensor in adult rat ventricular myocytes. *A,* fluorescence image of the CFP tagged type II regulatory (RII) subunit of the PKA-based biosensor in a control cell (-MβCD) and a cell treated with MβCD (+MβCD), as described in *Methods*. *B,* fluorescence intensity profile of CFP emission measured along the region highlighted (3 μm X 13 μm) in the control and MβCD-treated cells shown in panel *A*. The profile exhibited a sinusoidal pattern reflecting the concentration of the RII subunit along the Z-lines, as previously described (Warrier et al., 2007). *C,* average amplitude of the CFP fluorescence intensity maxima normalized to the minima in control (n = 8) and MβCD-treated (n = 7) cells (ns = not significant).

Figure S2. Cholesterol content of adult rat ventricular myocytes decreases with time in culture. Cholesterol was indexed in freshly isolated myocytes (Day 0) and myocytes maintained in culture for 24 (Day 1) or 72 hrs (Day 3) using confocal imaging of filipin-stained myocytes. Data in each group represents the average of 23-54 cells from three hearts. $p < 0.05$ for all pair-wise comparisons (ANOVA).

Figure S3. α-Cyclodextrin (αCD), an analog of methyl-β-cyclodextrin (MβCD) that does not bind cholesterol, does not alter the PKA-based biosensor response to β_1-adrenergic receptor stimulation. *A,* time course of changes in FRET response ($\Delta R/R_0$) and corresponding pseudocolor images recorded under control conditions *(a)*, and following exposure to 0.3 nM *(b)* and 30 nM Iso *(c)* in an αCD-treated myocyte. Scale bar, 10 μm. *B,* average changes in FRET responses to 0.3 nM Iso in untreated (control, n = 7), αCD-treated (n = 3) and MβCD-treated cells (n = 6) (** $p < 0.05$, ns = not significant). Treated cells were exposed to either 1 mM αCD or MβCD for 1 hr at 37°C in MEM-based culture medium as described in *Methods*.

Figure S4. The E-type prostaglandin receptor (EPR) agonist PGE1 does not stimulate the L-type Ca^{2+} current ($I_{Ca\text{-}L}$) in rat ventricular myocytes maintained in culture for 72 hrs.

A, time course of changes in $I_{Ca\text{-}L}$ amplitude and corresponding sample current traces (inset) under control conditions *(a)*, and following EPR activation with 10 μM PGE1 *(b)* and β-adrenergic receptor activation with 30 nM Iso (c). *B*, average increase in $I_{Ca\text{-}L}$ amplitude recorded in the presence of 10 μM PGE1 or 30 nM Iso. (** $p < 0.05$). Culture conditions and electrical recordings described in *Methods.*

Figure S1

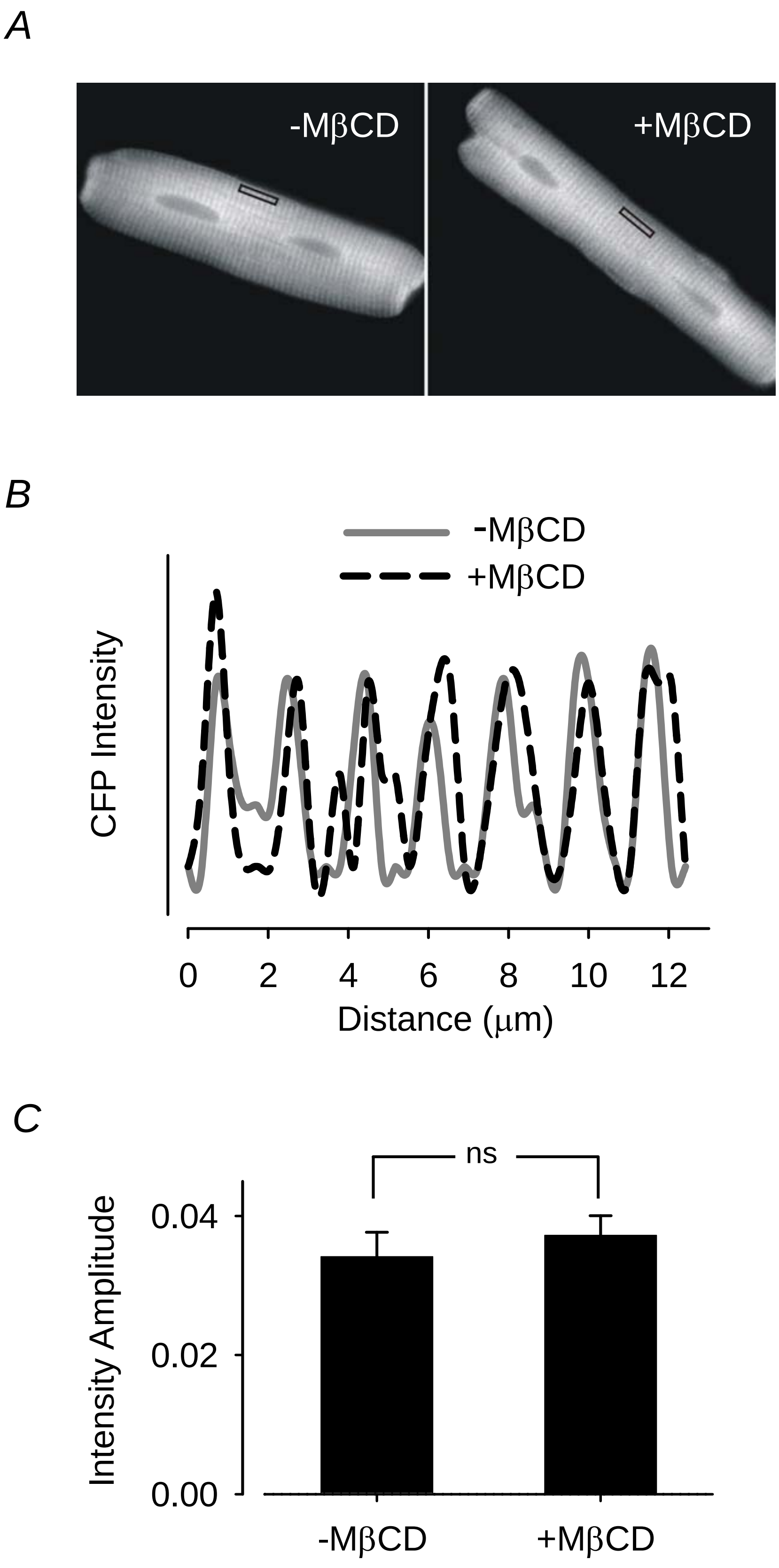
A
-MβCD
+MβCD
B
-MβCD
+MβCD
CFP Intensity
0
2
4
6
8
10
12
Distance (μm)
C
ns
Intensity Amplitude
0.04
0.02
0.00
-MβCD
+MβCD

Figure S2

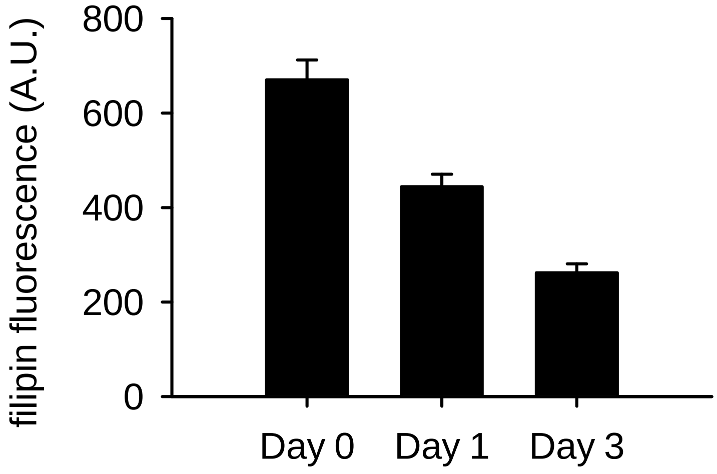

Figure S3

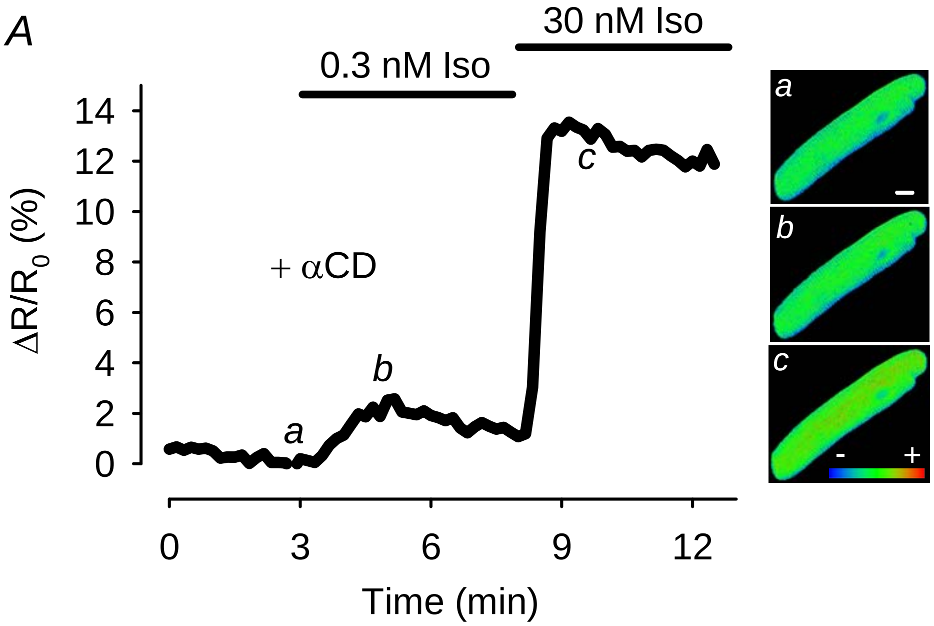
A
30 nM Iso
0.3 nM Iso
ΔR/R0 (%)
14
12
10
8
6
4
2
0
+ αCD
a
b
c
0
3
6
9
12
Time (min)
a
b
c
-
+

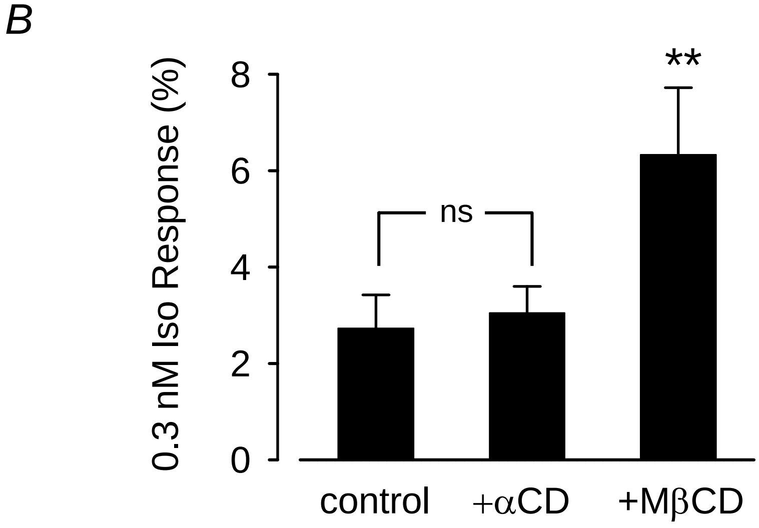
B
0.3 nM Iso Response (%)
8
6
4
2
0
**
ns
control
+αCD
+MβCD

Figure S4

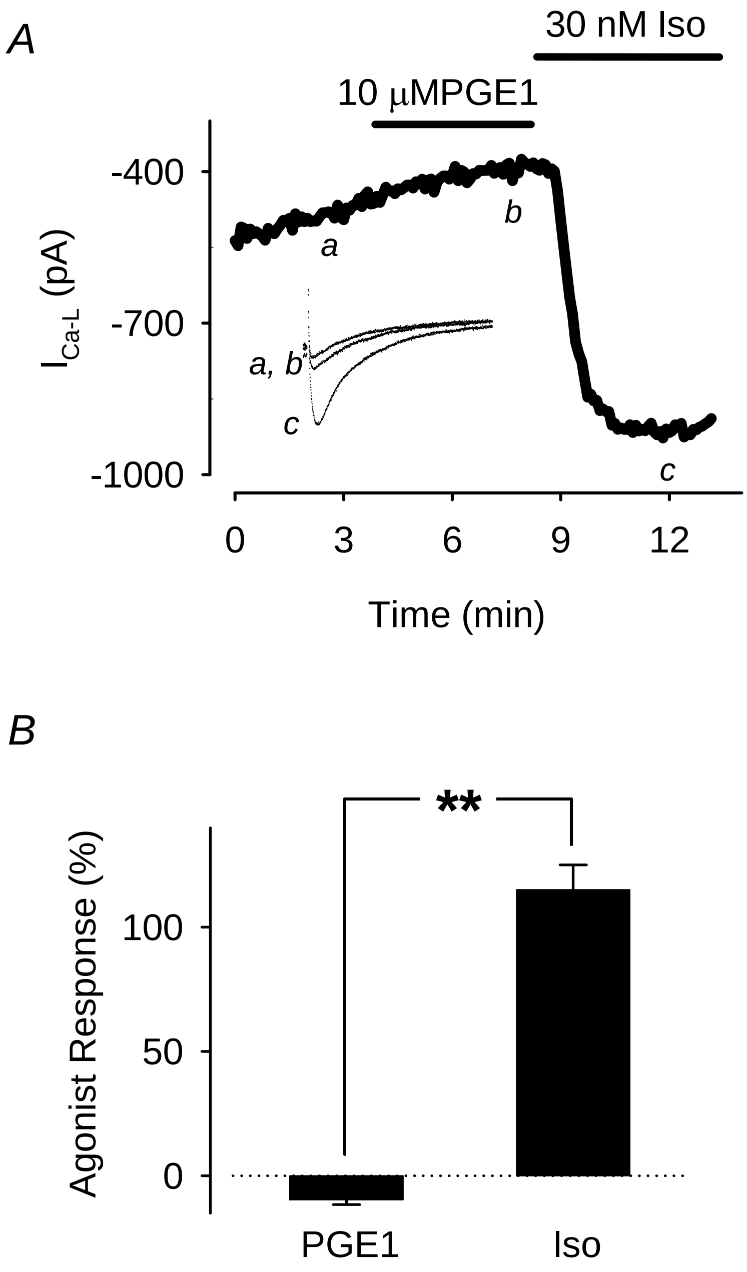
A
30 nM Iso
10 μMPGE1
-400
-700
-1000
I Ca-L (pA)
a
b
a, b
c
c
0
3
6
9
12
Time (min)
B
**
Agonist Response (%)
100
50
0
PGE1
Iso
